# Dynamic neutrophil-to-lymphocyte ratio as an independent prognostic marker of overall survival in patients receiving immune checkpoint inhibitors: a Latin American cohort study

**DOI:** 10.3389/fonc.2026.1930031

**Published:** 2026-09-17

**Authors:** Parag Chatterjee, Belén Insagaray, Tomás Ferraz, Mario González, Gianina Muñoz, Valeria Pombo, Romina Barrios, Mauricio Cuello

**Affiliations:** 1 Department of Biological Engineering, Universidad de la República, Paysandú, Uruguay; 2 Department of Clinical Oncology, Hospital de Clínicas, Montevideo, Uruguay; 3 Department of Mathematics and Statistics, Universidad de la República, Salto, Uruguay; 4 Instituto Nacional del Cáncer (National Cancer Institute), Montevideo, Uruguay

**Keywords:** cancer, ECOG performance status, immune checkpoint inhibitors, immunotherapy, neutrophil-to-lymphocyte ratio, prognostic biomarker, survival, time-varying analysis

## Abstract

**Background:**

The neutrophil-to-lymphocyte ratio (NLR) is an inexpensive, routinely available marker of systemic inflammation that has been proposed as a prognostic biomarker in cancer immunotherapy. However, most evidence derives from single baseline measurements in selected populations, and the prognostic value of *serial, on-treatment* NLR dynamics, particularly in real-world Latin American cohorts, remains poorly defined. We evaluated whether baseline and time-varying NLR predict overall survival (OS) in patients treated with immune checkpoint inhibitors (ICI), and whether any association is independent of performance status.

**Methods:**

We retrospectively analyzed 68 patients with advanced solid tumors (lung, urological, melanoma, and others) treated with ICI at two Uruguayan institutions: Hospital de Clínicas, Universidad de la República, and Instituto Nacional del Cáncer (INCA), both in Montevideo, Uruguay. NLR and platelet-to-lymphocyte ratio (PLR) were measured at baseline and at each treatment cycle (up to 12 cycles). OS and progression-free survival were analyzed using Kaplan–Meier estimates and Cox proportional-hazards models, including a time-varying covariate model using serial NLR. Six-month RECIST response and immune-related adverse events were assessed as secondary outcomes. Robustness was evaluated with bootstrap confidence intervals, permutation testing, and correction for multiple comparisons.

**Results:**

Over a median follow-up of 16.8 months, 39 deaths occurred (survival population n=66). Elevated baseline NLR was associated with shorter OS (HR 1.18 per unit, 95% CI 1.04–1.34, p=0.011). In a time-varying analysis using serial measurements, higher on-treatment NLR remained independently associated with OS after adjustment for ECOG performance status (HR 1.09, 95% CI 1.00–1.19, p=0.040; ECOG p=0.021). Patients in the highest NLR quartile (>4.9) had markedly shorter median OS than the remainder of the cohort (7.3 vs ~25 months). Neither baseline nor dynamic NLR/PLR predicted six-month RECIST response. Combination anti-PD-1/anti-CTLA-4 therapy was associated with higher odds of immune-related toxicity (OR 5.03, p=0.023).

**Conclusions:**

In this real-world Latin American ICI cohort, dynamic on-treatment NLR was independently associated with overall survival, supporting serial monitoring of this inexpensive marker over single baseline assessment. NLR did not predict tumor response, underscoring that its prognostic value reflects systemic host condition rather than direct antitumor activity. These findings, though exploratory, support NLR trajectory as a low-cost prognostic adjunct warranting prospective validation. Since complete blood counts are already obtained at every treatment cycle for routine hematologic surveillance, this information could be captured at no additional cost to clinical practice.

## 1 Introduction

Immune checkpoint inhibitors (ICI) targeting the programmed death-1 (PD-1), programmed death-ligand 1 (PD-L1), and cytotoxic T-lymphocyte-associated antigen-4 (CTLA-4) pathways have transformed the treatment of multiple advanced solid tumors, producing durable responses in a subset of patients across lung cancer, melanoma, and urological malignancies (1, 2, 27, 28). However, only a minority of patients derive long-term benefit, and a substantial proportion experience disease progression or clinically significant immune-related adverse events (irAEs) (3). The identification of inexpensive, widely available biomarkers that can stratify prognosis and inform treatment monitoring therefore remains a major unmet need in clinical immuno-oncology, particularly in resource-constrained and underrepresented healthcare settings (4).

The neutrophil-to-lymphocyte ratio (NLR), calculated from a routine complete blood count, has emerged as an attractive candidate marker of systemic inflammation and host immune competence (5). Elevated NLR is thought to reflect a pro-tumorigenic inflammatory milieu, characterized by neutrophil-driven immunosuppression and relative lymphocyte depletion, which may attenuate the antitumor immune response elicited by checkpoint blockade (6, 7). Platelets contribute a parallel, mechanistically distinct suppressive signal: they represent a major systemic source of transforming growth factor-β via the docking receptor GARP, directly impairing CD4+ and CD8+ T cell function, and platelet depletion has been shown to restore antitumor T cell activity in preclinical models (16). Neutrophils can further blunt immunotherapy efficacy through release of neutrophil extracellular traps (NETs), which have been linked to resistance to checkpoint blockade (17). Among the inflammatory indices proposed to date, NLR remains the most extensively validated: it is supported by an umbrella review synthesizing multiple systematic reviews and meta-analyses across tumor types (5), and its derived form (dNLR) constitutes the hematological component of the Lung Immune Prognostic Index, the most widely reproduced composite prognostic score in ICI-treated non-small cell lung cancer (18). Composite indices that additionally incorporate platelet counts, such as PLR or the systemic immune-inflammation index, are more susceptible to confounding by reactive thrombocytosis, chemotherapy-induced marrow suppression, and antiplatelet therapy, and have been less consistently reproduced across populations. NLR, in contrast, depends only on the neutrophil and lymphocyte counts already reported in every routine complete blood count, offering a directly interpretable, low-cost readout of the balance between innate immunosuppressive and adaptive effector compartments most relevant to checkpoint blockade efficacy. A growing body of evidence has associated high baseline NLR with poorer outcomes in ICI-treated patients, and the platelet-to-lymphocyte ratio (PLR) has been proposed as a complementary inflammatory index (8, 9). Nonetheless, the existing literature is dominated by retrospective analyses of single, pre-treatment measurements, frequently derived from selected cohorts in a limited number of geographic regions (10).

In current clinical practice, a complete blood count is already obtained at every treatment cycle to monitor for hematologic toxicity and guide dosing; the serial NLR values embedded in this routinely collected data are seldom analyzed beyond the cycle in which they were drawn. Two important gaps follow from this. First, ICI therapy is administered over repeated cycles, during which a patient’s inflammatory state evolves; a single baseline value cannot capture this trajectory. Whether *serial, on-treatment* NLR carries prognostic information beyond the baseline measurement has been far less studied, and methods that exploit longitudinally collected values, such as time-varying covariate survival models, are rarely applied (11). Second, NLR is strongly correlated with general clinical condition, and performance status is itself one of the most robust prognostic factors in oncology (12). Many reports associating NLR with survival have not formally tested whether this association is independent of the Eastern Cooperative Oncology Group (ECOG) performance status, leaving open the possibility that NLR is merely a surrogate for overall patient fitness (13).

Real-world data from Latin American populations are particularly scarce in this literature, despite the region’s distinct healthcare context and patient characteristics (14). To address these gaps, we conducted a retrospective cohort study of patients treated with ICI at two Uruguayan institutions, in which NLR and PLR were recorded at baseline and at every treatment cycle. Our primary objective was to evaluate the prognostic value of baseline and dynamic NLR for overall survival, with explicit assessment of whether any association is independent of ECOG performance status. As secondary objectives, we examined the relationship of these inflammatory markers with six-month radiological response and immune-related toxicity.

## 2 Methods

### 2.1 Study design and population

We conducted a retrospective cohort study of adult patients with advanced solid tumors treated with immune checkpoint inhibitors (ICI) at two Uruguayan institutions: Hospital de Clínicas, Universidad de la República, and Instituto Nacional del Cáncer (INCA), both in Montevideo, Uruguay. Consecutive patients who initiated ICI therapy and had at least a baseline complete blood count and documented clinical follow-up were eligible. Patients received anti-PD-1 monotherapy, anti-PD-L1 monotherapy, or combination anti-PD-1/anti-CTLA-4 therapy, according to institutional protocols and approved indications. The study was conducted in accordance with the Declaration of Helsinki and approved by the institutional ethics committee [n 60-24]; the requirement for individual informed consent was waived given the retrospective use of de-identified clinical data.

### 2.2 Clinical variables and outcomes

Baseline clinical variables included age, sex, treating institution, tumor type, ICI regimen, line of therapy, and ECOG performance status at ICI initiation (25). Complete blood counts—including absolute neutrophil, lymphocyte, and platelet counts—were recorded at baseline (cycle 1) and at each subsequent treatment cycle, up to 12 cycles. The NLR was calculated as the absolute neutrophil count divided by the absolute lymphocyte count, and the PLR as the absolute platelet count divided by the absolute lymphocyte count, at each available timepoint.

The primary outcome was overall survival (OS), defined as the time from ICI initiation to death from any cause; patients alive at last follow-up were censored at that date. Progression-free survival (PFS) was defined as the time from ICI initiation to radiologically confirmed progression or death, with censoring at last follow-up for patients without an event. Secondary outcomes were radiological response at six months, assessed by the treating teams according to RECIST 1.1 criteria (26) using the nearest available imaging study and dichotomized as response versus progression, and immune-related adverse events (irAEs), recorded by type and, where available, graded as mild (CTCAE < 2) or significant (CTCAE ≥ 2). Grades corresponded to those documented in the clinical chart when explicitly recorded at the time of occurrence; when not explicitly graded, irAEs were graded retrospectively by the study team at the time of data review, based on the available laboratory and clinical findings.

### 2.3 Analysis populations

To avoid loss of information, analysis populations were defined per outcome. The survival population comprised all patients with a treatment start date and follow-up information. The response-evaluable population comprised patients with a six-month RECIST assessment. The toxicity-evaluable population comprised all patients with documented irAE status. One patient with a recorded progression date preceding the treatment start date (a data-entry inconsistency) was excluded from PFS analyses only and retained for all other analyses.

### 2.4 Feature engineering

For each patient, baseline NLR and PLR were defined from cycle 1. Dynamic features were derived from the serial measurements, including absolute and percentage change between cycles, the mean and slope of NLR over the first three cycles, and the cycle-3-to-cycle-1 ratio. Where a derived ratio was missing but the underlying neutrophil, lymphocyte, and platelet counts were available, the ratio was recomputed from the component counts. Non-numeric annotations in the source data (e.g., notations indicating that a sample was not obtained) were treated as missing values.

### 2.5 Statistical analysis

Cohort characteristics were summarized using medians with interquartile ranges or ranges for continuous variables and counts with percentages for categorical variables. OS and PFS were estimated using the Kaplan–Meier method (20), and survival distributions across NLR strata were compared using the log-rank test (21). The prognostic value of baseline and clinical covariates was assessed using Cox proportional-hazards models (19), reported as hazard ratios (HR) with 95% confidence intervals (CI). Candidate predictors were first examined univariately; multivariable models then adjusted the baseline NLR association for ECOG performance status, age, and line of therapy, with the number of events per variable monitored to limit overfitting.

To exploit the serial measurements, a time-varying covariate Cox model was fitted in which NLR was updated at each treatment cycle, modeled in a counting-process (start–stop) format, both unadjusted and adjusted for ECOG performance status. Extreme NLR values were capped at the 99th percentile to limit the influence of outliers. To assess whether the prognostic association was consistent across the tumor types represented in the cohort, the time-varying model was repeated with adjustment for tumor type, a tumor-type × NLR interaction term was tested, and tumor-type-specific models were fitted for descriptive purposes.

For the NLR threshold analysis, pre-specified clinical cutoffs (NLR > 3, NLR > 5, and the cohort median) were evaluated to avoid data-driven cutpoint selection. A maximally selected log-rank statistic was additionally computed, with the resulting p-value corrected for multiple cutpoint testing using the approximation of Lausen and Schumacher (22); survival was also summarized across NLR quartiles.

Associations of baseline and dynamic markers with six-month response and with toxicity were assessed using the Mann–Whitney U test for continuous variables and Fisher’s exact test for categorical variables. As a secondary, exploratory analysis, supervised machine-learning models (logistic regression, random forest, gradient boosting, and support vector machines) were evaluated for response prediction using stratified five-fold cross-validation with synthetic minority oversampling applied within training folds; discrimination was summarized by the area under the receiver-operating-characteristic curve (AUC) (23). To guard against spurious findings, the robustness of key results was assessed using bootstrap confidence intervals, permutation testing against label-shuffled null distributions, and correction for multiple comparisons using the Benjamini–Hochberg false-discovery-rate procedure (24).

All analyses were performed in Python (version 3.x) using the pandas, scikit-learn, imbalanced-learn, lifelines, and statsmodels libraries. A two-sided p-value < 0.05 was considered statistically significant, with appropriate caution applied to exploratory analyses involving multiple comparisons.

## 3 Results

### 3.1 Cohort characteristics

A total of 68 patients met the inclusion criteria (**Table 1**). The median age was 64 years (range 25–87), and 43 (63%) were male. The most frequent tumor types were lung (n=24, 35%), urological (n=21, 31%), and skin/melanoma (n=19, 28%). Most patients received anti-PD-1 monotherapy (n=44, 65%), while 13 (19%) received combination anti-PD-1/anti-CTLA-4 therapy and 11 (16%) received anti-PD-L1 monotherapy. ECOG performance status at ICI initiation was 0 in 14 patients, 1 in 40, and 2–3 in 12; it was not recorded in 2. ICI was administered as first-line therapy in 49 patients (72%). The median baseline NLR was 2.99 (interquartile range [IQR] 2.45–4.84) and the median baseline PLR was 153.9 (IQR 114.4–214.3). The distribution of clinical and demographic characteristics is summarized in **Figure 1**.

**Table 1:** Baseline characteristics of the study cohort (N = 68).

Characteristic	Value
Age, years — median (range)	64 (25–87)
Sex — n (%)	
Male	43 (63)
Female	25 (37)
Treating institution — n (%)	
Hospital de Clínicas	42 (62)
Instituto Nacional del Cáncer (INCA)	26 (38)
Tumor type — n (%)	
Lung	24 (35)
Urological	21 (31)
Skin/melanoma	19 (28)
Colorectal	3 (4)
Head and neck	1 (1)
ICI regimen — n (%)	
Anti-PD-1 monotherapy	44 (65)
Anti-PD-1 + anti-CTLA-4	13 (19)
Anti-PD-L1 monotherapy	11 (16)
ECOG performance status — n (%)	
0	14 (21)
1	40 (59)
2–3	12 (18)
Not recorded	2 (3)
Line of therapy — n (%)	
First line	49 (72)
Second line or later	17 (25)
Not recorded	2 (3)
Baseline NLR — median (IQR)	2.99 (2.45–4.84)
Baseline PLR — median (IQR)	153.9 (114.4–214.3)
Six-month response (evaluable n = 62) — n (%)	
Response	43 (69)
Progression	19 (31)
Immune-related toxicity (n = 68) — n (%)	
Any irAE	26 (38)
Significant (CTCAE ≥ 2)	11 (16)

NLR, neutrophil-to-lymphocyte ratio; PLR, platelet-to-lymphocyte ratio; ICI, immune checkpoint inhibitor; PD-1, programmed death-1; PD-L1, programmed death-ligand 1; CTLA-4, cytotoxic T-lymphocyte-associated antigen-4; ECOG, Eastern Cooperative Oncology Group; irAE, immune-related adverse event; CTCAE, Common Terminology Criteria for Adverse Events; IQR, interquartile range. Percentages are rounded and may not sum to 100. Six-month response was evaluable in 62 patients with an available RECIST assessment.

**Figure 1:**
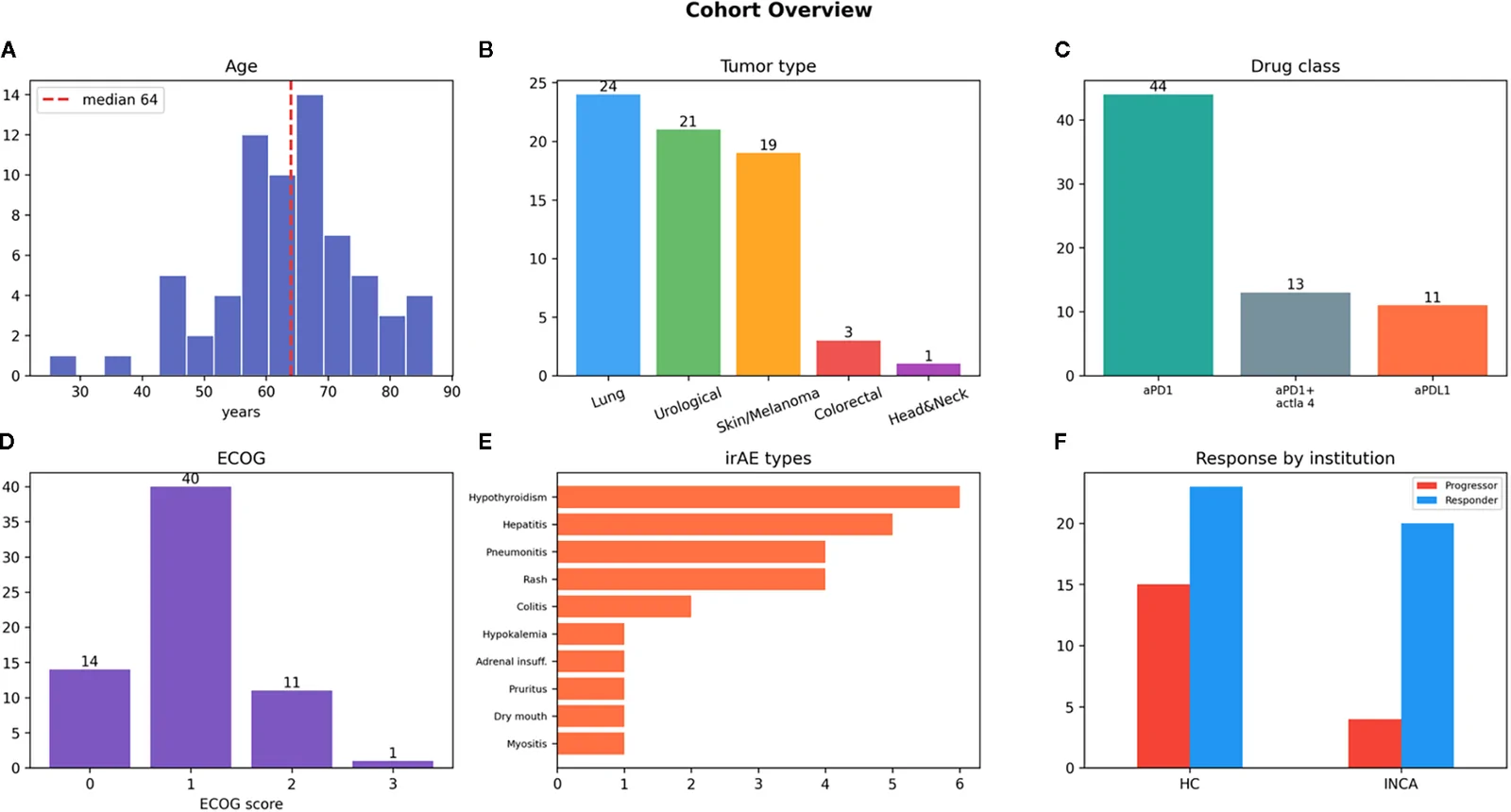
Overview of the study cohort. Distribution of **(A)** age, **(B)** tumor type, **(C)** immune checkpoint inhibitor regimen, **(D)** ECOG performance status at treatment initiation, **(E)** types of immune-related adverse events, and **(F)** six-month radiological response stratified by treating institution. ECOG, Eastern Cooperative Oncology Group; PD-1, programmed death-1; PD-L1, programmed death-ligand 1; CTLA-4, cytotoxic T-lymphocyte-associated antigen-4.

### 3.2 Overall and progression-free survival

Over a median follow-up of 16.8 months, 39 deaths occurred in the survival population (n=66). The median OS was 21.9 months and the median PFS was 14.6 months (45 events).

In univariable Cox analysis of OS, ECOG performance status was the strongest prognostic factor (HR 3.24, 95% CI 1.87–5.60, p<0.001). Higher baseline NLR was significantly associated with shorter OS (HR 1.18 per unit increase, 95% CI 1.04–1.34, p=0.011; Table 2), as was the mean NLR over the first three cycles (HR 1.16, 95% CI 1.00–1.34, p=0.048). Baseline PLR, age, sex, line of therapy, and treating institution were not significantly associated with OS (all p>0.05). In univariable analysis of PFS, ECOG performance status was the only significant predictor (HR 2.01, 95% CI 1.24–3.27, p=0.005); no other predictor reached significance.

### 3.3 Baseline NLR and overall survival are confounded by performance status

In a multivariable model adjusting baseline NLR for ECOG performance status, age, and line of therapy, ECOG remained strongly and independently associated with OS (HR 2.83, 95% CI 1.61–4.99, p<0.001), whereas the association of baseline NLR was attenuated and no longer statistically significant (HR 1.11, 95% CI 0.98–1.26, p=0.107; concordance index 0.728). The attenuation was confirmed by bootstrap resampling: the OS hazard ratio for baseline NLR was 1.18 (95% CI 1.02–1.40) univariately but 1.09 (95% CI 0.96–1.33) after adjustment for ECOG. These findings indicate that the prognostic signal of a single baseline NLR measurement is substantially explained by general performance status.

### 3.4 Dynamic on-treatment NLR is independently associated with overall survival

To exploit the serially collected measurements, we fitted a time-varying covariate Cox model in which NLR was updated at each treatment cycle (541 measurement intervals across 66 patients, 39 events). Higher on-treatment NLR was associated with shorter OS in the unadjusted model (HR 1.11 per unit, 95% CI 1.02–1.20, p=0.020). Critically, this association persisted after adjustment for ECOG performance status (NLR HR 1.09, 95% CI 1.00–1.19, p=0.040; ECOG HR 1.48, 95% CI 1.06–2.06, p=0.021), indicating that the dynamic, on-treatment trajectory of NLR carries prognostic information beyond both the single baseline value and the patient’s performance status (**Figure 2**).

**Figure 2:**
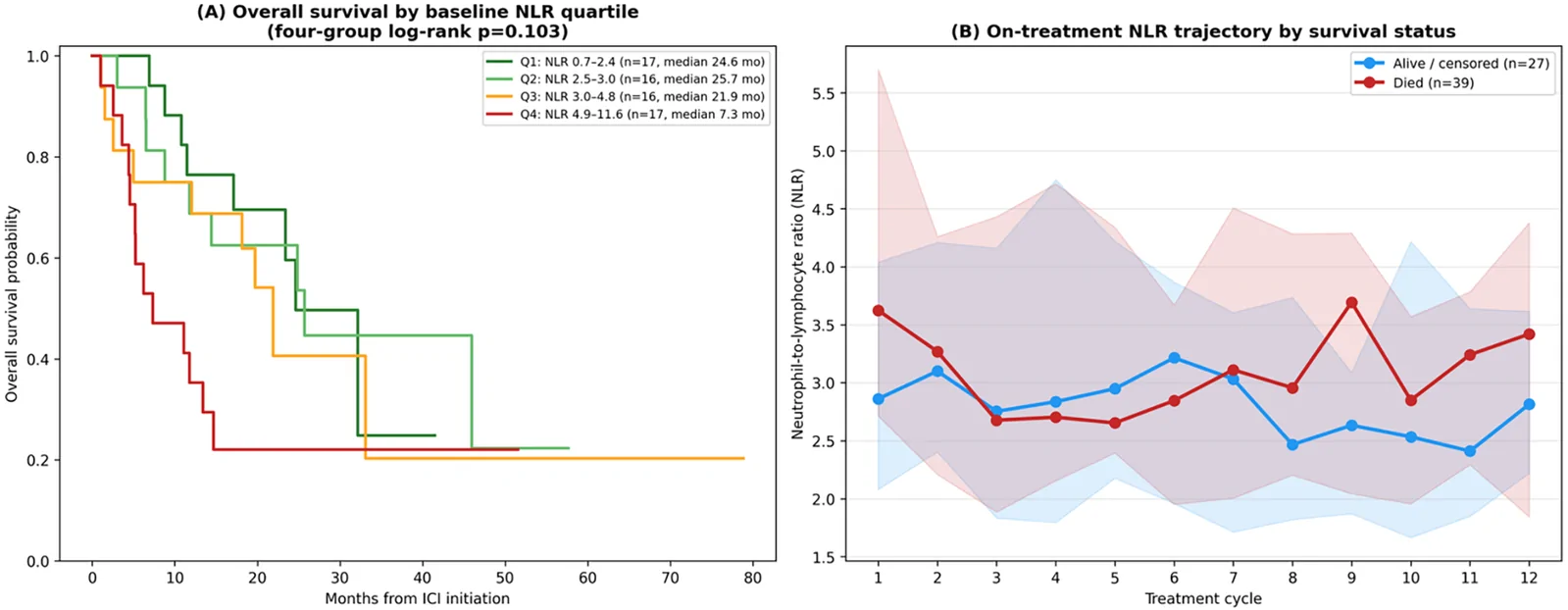
Neutrophil-to-lymphocyte ratio (NLR) and overall survival. **(A)** Kaplan–Meier estimates of overall survival stratified by pre-specified baseline NLR quartiles. Patients in the highest quartile (Q4; NLR 4.9–11.6) had markedly shorter median overall survival (7.3 months) than those in the lower three quartiles (medians 21.9–25.7 months), which were similar to one another; the four-group log-rank test was not statistically significant (p = 0.103), consistent with a prognostic effect concentrated at the upper end of the NLR distribution. **(B)** Median on-treatment NLR at each treatment cycle for patients who died (red) versus those alive or censored at last follow-up (blue); shaded areas represent the interquartile range, and only cycles with at least five evaluable patients per group are shown. Patients who died had higher baseline NLR and higher NLR in later cycles, whereas survivors showed a gradual decline. The number of evaluable patients decreased in later cycles; late-cycle estimates are descriptive and complement the time-varying Cox analysis (**Table 2**). NLR, neutrophil-to-lymphocyte ratio; ICI, immune checkpoint inhibitor.

**Table 2:** Cox proportional-hazards models for overall survival.

Predictor	HR	95% CI	p
Univariable models			
ECOG performance status	3.24	1.87–5.60	<0.001
Baseline NLR (per unit)	1.18	1.04–1.34	0.011
Mean NLR, cycles 1–3 (per unit)	1.16	1.00–1.34	0.048
Baseline PLR (per unit)	1.00	1.00–1.00	0.157
Age (per year)	0.99	0.96–1.02	0.436
Line of therapy (≥2 vs 1)	0.56	0.25–1.23	0.148
Treating institution	0.97	0.51–1.85	0.933
Multivariable model (baseline NLR)			
Baseline NLR (per unit)	1.11	0.98–1.26	0.107
ECOG performance status	2.83	1.61–4.99	<0.001
Age (per year)	0.98	0.95–1.01	0.166
Line of therapy (≥2 vs 1)	0.52	0.23–1.18	0.117
Time-varying model (serial NLR)			
On-treatment NLR, unadjusted (per unit)	1.11	1.02–1.20	0.020
On-treatment NLR, adjusted for ECOG	1.09	1.00–1.19	0.040
ECOG (in adjusted time-varying model)	1.48	1.06–2.06	0.021

Hazard ratios for baseline NLR, PLR, mean NLR, and age are expressed per one-unit increase. The multivariable baseline model included 66 patients with 39 events (concordance index = 0.728). The time-varying model used 541 measurement intervals across 66 patients (39 events), with NLR updated at each treatment cycle and capped at the 99th percentile to limit the influence of outliers. HR, hazard ratio; CI, confidence interval; NLR, neutrophil-to-lymphocyte ratio; PLR, platelet-to-lymphocyte ratio; ECOG, Eastern Cooperative Oncology Group.

### 3.5 NLR thresholds and survival stratification

Patients with markedly elevated NLR survived, on median, only about a quarter as long as the remainder of the cohort. Using pre-specified clinical cutoffs, patients with a baseline NLR > 5 had a shorter median OS than those with NLR ≤ 5 (7.3 versus 24.9 months), although this did not reach statistical significance (log-rank p=0.059); a similar pattern was observed at the NLR > 3 and median cutoffs (p=0.071). A data-driven optimal cutpoint (NLR > 4.25) yielded an uncorrected log-rank p=0.009, but after correction for multiple cutpoint testing this was no longer significant (corrected p=0.100), underscoring the absence of a robust single dichotomizing threshold. Analysis by NLR quartile was more informative: patients in the highest quartile (NLR 4.9–11.6) had a markedly shorter median OS of 7.3 months, compared with 22–26 months across the lower three quartiles (**Figure 2**), indicating that the prognostic deterioration is concentrated at the upper end of the NLR range rather than reflecting a discrete cutpoint.

### 3.6 Inflammatory markers do not predict radiological response

In the response-evaluable population (n=62; 43 responders, 19 progressors), neither baseline NLR (responder median 3.01 versus progressor median 2.97, p=0.604) nor baseline PLR (153.9 versus 166.2, p=0.698) differed significantly between response groups. In a secondary, exploratory machine-learning analysis undertaken only to test whether multivariable combinations of inflammatory features carried response information not evident univariately, acknowledged as underpowered in a cohort of this size, models using baseline or dynamic inflammatory features achieved only modest discrimination for six-month response (highest AUC 0.63), and this did not exceed chance under permutation testing (p=0.159). No individual feature was significantly associated with response after false-discovery-rate correction. Thus, NLR and PLR did not predict tumor response in this cohort.

### 3.7 Immune-related toxicity

Immune-related adverse events occurred in 26 of 68 patients (38%), of which 11 were graded as significant (CTCAE ≥ 2). The most frequent irAEs were endocrine, hepatic, and dermatological. Neither baseline NLR nor early NLR dynamics were significantly associated with the occurrence of toxicity (all p>0.05), and no feature survived false-discovery-rate correction. Combination anti-PD-1/anti-CTLA-4 therapy was associated with substantially higher odds of immune-related toxicity compared with monotherapy (odds ratio 5.03, Fisher’s exact p=0.023), consistent with the established toxicity profile of combination checkpoint blockade.

### 3.8 Early neutrophil dynamics and immune-related toxicity

Given the biological rationale that immune activation following the first ICI infusion may manifest as early changes in circulating myeloid and lymphoid populations, we examined the absolute leukocyte counts and their early on-treatment dynamics in relation to irAE occurrence. The relative change in absolute neutrophil count from cycle 1 to cycle 2 was significantly associated with subsequent immune-related toxicity: patients who developed irAEs showed a decline in absolute neutrophils after the first infusion (median −10.1%), whereas those without irAEs showed an increase, statistically significant (median +11.2%; Mann–Whitney U, p=0.045). A parallel trend was observed for the absolute change in neutrophil count (p=0.074) and for platelet dynamics (p=0.087), though these did not reach significance. This early neutrophil signal contrasts with the ratio-based measures (baseline and dynamic NLR/PLR), none of which were associated with toxicity. While this association did not persist after correction for multiple comparisons across the full panel of exploratory count-based features, its biological coherence and directionality make it a noteworthy signal warranting dedicated prospective evaluation.

### 3.9 Sensitivity analyses: tumor-type heterogeneity and additional prognostic factors

Because the cohort included several tumor types, we assessed whether the prognostic association was consistent across them. Overall survival did not differ significantly by tumor type (log-rank p=0.300), supporting analysis of the pooled cohort. The association between time-varying NLR and overall survival was unchanged after adjustment for tumor type (HR 1.09, 95% CI 1.01–1.19, p=0.039), and no NLR × tumor-type interaction was detected (all p≥0.180). Tumor-type-specific models were directionally consistent (all HR ≥1.00) but individually underpowered, with only 9–15 events per group (lung HR 1.06, p=0.209; urological HR 1.00, p=0.911; melanoma HR 1.01, p=0.850).

## 4 Discussion

In this real-world cohort of 68 patients treated with immune checkpoint inhibitors at two Uruguayan institutions, we found that the dynamic, on-treatment neutrophil-to-lymphocyte ratio was independently associated with overall survival, whereas a single baseline NLR measurement was not, once performance status was taken into account. To our knowledge, this is among the first studies to apply a time-varying covariate survival model to serial NLR measurements in an ICI-treated population from a Latin American cohort. Our findings suggest that the *trajectory* of this inexpensive, routinely available marker may be more informative than its starting value alone, although this observation requires confirmation before it can be considered clinically actionable.

Our results help reconcile an apparent tension in the existing literature. Numerous studies have reported that elevated baseline NLR predicts poorer outcomes in patients receiving checkpoint blockade (5, 8, 9). We reproduced this association in univariable analysis but found that it was substantially attenuated and lost statistical significance after adjustment for ECOG performance status. This is consistent with the recognized correlation between systemic inflammation and general clinical condition, and with the observation that many prior NLR studies did not formally adjust for performance status (12, 13). Our finding suggests that part of the prognostic value attributed to baseline NLR in earlier reports may reflect its role as a surrogate for patient fitness rather than an independent biological signal. Importantly, however, the time-varying analysis demonstrated that on-treatment NLR retained an independent association with survival even after this adjustment—indicating that serial NLR captures evolving disease- and host-related biology that is not reduced to baseline performance status.

The mechanistic interpretation is consistent with current understanding of NLR as a marker of the systemic inflammatory and immunosuppressive milieu (6, 7). A rising on-treatment NLR may reflect progressive neutrophil-driven immunosuppression, lymphocyte exhaustion or depletion, or subclinical disease progression, each of which would be expected to track with worsening prognosis. The concentration of risk in the highest NLR quartile, with a median OS of only 7.3 months, supports the view that markedly elevated systemic inflammation identifies a particularly poor-prognosis subgroup, even though we found no robust single dichotomizing threshold. This gradient pattern argues against rigid cutoff-based use of NLR and in favor of interpreting it as a continuous, serially monitored marker.

From a practical standpoint, our findings suggest that the complete blood counts already obtained at each treatment cycle for routine hematologic toxicity monitoring could be leveraged, at no additional cost, to flag patients with a rising NLR trajectory for closer clinical surveillance, particularly within the highest NLR range, where survival deterioration was concentrated. This does not yet support using NLR to withhold or alter ICI therapy, given its modest discrimination for treatment response, but it may help prioritize which patients warrant more frequent clinical assessment or earlier investigation of subclinical disease progression. Practically, this could be implemented without new infrastructure: because dynamic NLR is derived entirely from the differential complete blood count already performed before every cycle, no additional sampling, assay, or cost is required. Implementation would consist only of computing and recording a ratio the analyzer already reports, ideally as an automatically calculated field in the laboratory information system.

An exploratory but biologically coherent signal emerged linking early neutrophil dynamics to immune-related toxicity: patients who subsequently developed irAEs showed a decline in absolute neutrophil count after the first infusion, rather than the increase seen in patients without toxicity. This is consistent with reports that on-treatment leukocyte kinetics track the onset of immune activation; in a larger cohort (15), it was found that NLR dynamics preceded and predicted irAE onset and severity, including NLR elevation weeks before symptomatic pneumonitis. That the signal in our cohort was carried by the absolute neutrophil count rather than the ratio suggests that component counts may retain information lost in ratio normalization, and merits dedicated investigation in adequately powered, adverse-event-specific cohorts.

In contrast to its prognostic value for survival, NLR did not predict radiological response at six months, and exploratory machine-learning models did not exceed chance for this task. With only 62 response-evaluable patients these models are underpowered, and they are reported solely as an exploratory result. This dissociation is itself informative: it suggests that NLR reflects host condition and global prognosis rather than the specific likelihood of tumor shrinkage, and it cautions against using inflammatory ratios as predictors of response in clinical decision-making. The strong association we observed between combination anti-PD-1/anti-CTLA-4 therapy and immune-related toxicity is consistent with the well-established toxicity profile of combination checkpoint blockade (3) and supports the internal validity of our dataset, while no inflammatory marker independently predicted toxicity.

This study has several limitations that warrant caution. First, it is a retrospective analysis from two centers with a modest sample size, and the survival findings—while internally robust to bootstrap resampling and adjustment—are based on a limited number of events and should be considered exploratory and hypothesis-generating. The cohort of 68 patients corresponds to all ICI-treated patients meeting eligibility criteria at the two participating centers during the study period, rather than a selected subsample, reflecting the limited number of ICI-treated patients at referral centers in a small country. This gives the cohort unusual longitudinal depth relative to its size, which is what makes the time-varying analysis feasible, but statistical power remains limited and bootstrap resampling does not substitute for external validation in an independent cohort, which was not performed here. Second, response was assessed by treating teams using the nearest available imaging rather than by blinded central review, and toxicity grading was simplified to a binary classification, introducing potential measurement variability. Third, although the association was unchanged after adjustment for tumor type and no interaction was detected, the cohort was underpowered for tumor-specific inference. Fourth, we did not have complete data on other established prognostic factors such as lactate dehydrogenase, C-reactive protein, tumor burden, PD-L1 expression, or corticosteroid exposure; with 39 events, adding such covariates would exceed what the sample can support, and independence from this wider set of confounders remains to be established. NLR elevation is also not specific to checkpoint-related immune activation: intercurrent infection, corticosteroid use, and tumor necrosis can all raise it, and none could be adjudicated at each measurement in this retrospective dataset. Finally, because serial values are available only while patients remain on treatment, those with early progression or death contribute fewer measurements, raising the possibility of immortal time bias and informative missingness. The counting-process formulation attributes risk only to intervals during which patients were under observation, which mitigates but does not eliminate this concern.

Notwithstanding these limitations, our study has notable strengths, including the use of serially collected measurements analyzed with an appropriate time-varying model, explicit adjustment for performance status, rigorous statistical safeguards against spurious findings (bootstrap confidence intervals, permutation testing, and correction for multiple comparisons), and the contribution of real-world data from an underrepresented Latin American population. These features distinguish the present analysis from much of the existing NLR literature, in which single baseline measurements and limited confounder adjustment predominate.

In conclusion, in this real-world Latin American ICI cohort, dynamic on-treatment NLR was independently associated with overall survival, whereas baseline NLR was confounded by performance status and neither marker predicted tumor response. These findings suggest that serial monitoring of NLR could represent a low-cost prognostic adjunct during checkpoint inhibitor therapy, pending validation in larger, multicenter prospective cohorts. If confirmed, dynamic NLR could contribute to longitudinal risk stratification in immuno-oncology, particularly in resource-constrained settings where more expensive biomarkers are unavailable.

## Data Availability

The data analyzed in this study is subject to the following licenses/restrictions: Access to the dataset is restricted to protect patient privacy and confidentiality, given the retrospective clinical nature of the data and the small size of the cohort, which increases the risk of patient re-identification even after de-identification. The ethics approval covered retrospective analysis of de-identified clinical data for the purposes of this study. De-identified data may be made available by the corresponding author upon reasonable request, subject to institutional approval and, if required, a data-sharing agreement with Hospital de Clínicas and Instituto Nacional del Cáncer (National Cancer Institute), Montevideo, Uruguay. Requests to access these datasets should be directed to Dr. Parag Chatterjee, paragc@ieee.org & Dr. Belén Insagaray, belen.insagaray@gmail.com.
